# Global Bivariate Meta‐Analysis of FIB‐4 Cut‐Offs to Rule Out Advanced Fibrosis in MASLD

**DOI:** 10.1155/ijh/9419416

**Published:** 2026-01-14

**Authors:** Anuradha Ghosal, Samit Ghosal

**Affiliations:** ^1^ Department of Medical Sciences, Aston Medical School, Aston University, Birmingham, UK, aston.ac.uk; ^2^ Department of Endocrinology, Nightingale Hospital, Kolkata, West Bengal, India, nightingalehospital.com

**Keywords:** advanced liver fibrosis, FIB-4 index, MASLD, meta-analysis, non-invasive tests

## Abstract

**Background:**

The fibrosis‐4 (FIB‐4) index, a non‐invasive marker, evaluates advanced fibrosis in metabolic dysfunction‐associated steatotic liver disease (MASLD), but the variability in its performance across different populations remains unclear.

**Methods:**

We conducted a systematic review and bivariate meta‐analysis of 14 studies (*N* = 5521) with 2 × 2 contingency data for FIB‐4 at thresholds < 1.0 and < 1.3, compared with liver biopsy or transient elastography (TE). The Reitsma model (R v4.4.3) estimated pooled sensitivity and specificity. Subgroup analyses assessed region (India vs. global) and reference standard effects, with heterogeneity and publication bias (Deeks′ test) evaluated.

**Results:**

Pooled sensitivity was 0.73 (95% CI: 0.69–0.77), and specificity was 0.69 (95% CI: 0.61–0.76) at < 1.3. The < 1.0 threshold demonstrated higher specificity (0.83, 95% CI: 0.75–0.89) but lower sensitivity (0.63, 95% CI: 0.58–0.68). Indian cohorts (*n* = 3) exhibited higher specificity (0.83) than the global estimate (0.66) at < 1.3, whereas sensitivity remained similar.

**Conclusion:**

FIB‐4 below 1.3 is a useful initial tool for ruling out advanced fibrosis in MASLD, with potential regional variations in specificity. Larger studies are needed to confirm cut‐offs, supporting tailored guidelines with sequential testing.

## 1. Introduction

Metabolic dysfunction‐associated steatotic liver disease (MASLD) affects up to one‐third of the global population and is closely linked to metabolic risk factors such as Type 2 diabetes and obesity [1]. The presence of advanced fibrosis (≥F3) in MASLD predicts progression to cirrhosis and hepatocellular carcinoma, emphasising the importance of early and reliable detection [2]. Although liver biopsy remains the gold standard, its invasiveness and susceptibility to sampling errors have led to the increased adoption of non‐invasive tests (NITs) [3].

Among these, the fibrosis‐4 (FIB‐4) index, which is based on age, AST, ALT and platelet count, is widely used because of its low cost and convenience [4]. Typically, a cut‐off of > 1.3 is used to exclude ≥F3, with an upper threshold of > 2.67 indicating a high risk [4]. However, the diagnostic performance of FIB‐4 varies across populations. This variability is likely driven by differences in age (because age is a component of the FIB‐4 formula), the burden and pattern of metabolic risk factors (including Type 2 diabetes, obesity and other cardiometabolic comorbidities), underlying ethnic and regional risk profiles for MASLD and methodological factors such as referral‐centre versus population‐based sampling and the choice of reference standard (liver biopsy vs. transient elastography [TE]). These factors may alter both the pretest probability of ≥F3 and the operating characteristics of FIB‐4, leading to genuine between‐study heterogeneity. The validity of lower cut‐off remains uncertain, especially in high‐risk groups such as individuals with diabetes, where FIB‐4 may be less predictive. Notably, recent Indian studies, including the MISHTI trial, have reported limited concordance between FIB‐4 and TE, raising concerns regarding its clinical utility in local settings [5]. On this basis, we prespecified region (Indian vs. non‐Indian/global cohorts), FIB‐4 cut‐off (< 1.0 vs. < 1.3) and reference standard (histology vs. TE) as potential effect modifiers. The < 1.0 and < 1.3 thresholds were prespecified a priori, informed by existing clinical practice guidelines and previous meta‐analyses. The < 1.3 cut‐off is widely endorsed by AASLD (2023) and EASL (2021) clinical practice guidance as the standard rule out value for ≥F3 in MASLD. A lower < 1.0 threshold has been proposed in recent Asian and younger metabolic cohorts to enhance sensitivity and reduce missed cases in populations with lower pretest probability [6]. Importantly, no post hoc threshold optimisation was performed in this analysis to avoid data‐driven bias. These variables were used as subgroup and meta‐regression covariates to formally assess whether the hypothesised sources of heterogeneity influence the sensitivity and false positive rate (FPR) of FIB‐4 in MASLD.

This systematic review and meta‐analysis aimed to assess the diagnostic accuracy of the FIB‐4 index in detecting ≥F3 in patients with MASLD. It specifically investigates the influence of cut‐off thresholds (< 1.0 vs. < 1.3), regional variations (India vs. global) and reference standards (histology vs. TE). Sensitivity analyses, publication bias evaluation and QUADAS‐2 risk of bias assessment were conducted to guide evidence‐based clinical decisions.

## 2. Materials and Methods

### 2.1. Study Design

This systematic review and meta‐analysis adhered to PRISMA 2020 guidelines and was prospectively registered in the PROSPERO database (Registration ID: CRD420251105536) [7].

### 2.2. PICO Framework

To guide the study selection and synthesis, the PICO framework was applied:

• Population (P): adults (≥ 18 years) diagnosed with MASLD.
• Index Test (I): FIB‐4 score with cutoffs of < 1.0 and < 1.3.
• Comparator (C): reference standards—liver biopsy (histology) or TE.
• Outcomes (O): diagnostic accuracy metrics for identifying ≥F3, including sensitivity, specificity, area under the curve (AUC) and FPR.

Advanced fibrosis was defined as ≥F3 on histology or the corresponding threshold on TE, consistent with MASLD diagnostic criteria and prior meta‐analytic methodology. For studies using TE as the reference standard, fibrosis staging was based on liver stiffness measurement (LSM) cut‐offs as defined in the original publications. These thresholds varied across studies according to population characteristics and local validation, but generally ranged between approximately 8–10 kPa for ≥F3 and ≥ 12–14 kPa for cirrhosis (F4). The present meta‐analysis did not impose a single LSM cut‐off. Controlled attenuation parameter (CAP) values were not used to define fibrosis stage, as CAP reflects hepatic steatosis rather than fibrosis severity. Stage‐specific performance (e.g., separate F3 vs. F4 estimates) was not analysed because most included studies reported only binary outcomes for advanced versus non‐≥F3 rather than fibrosis‐stage–specific diagnostic accuracy.

## 3. Literature Search Strategy

**Figure 1:**
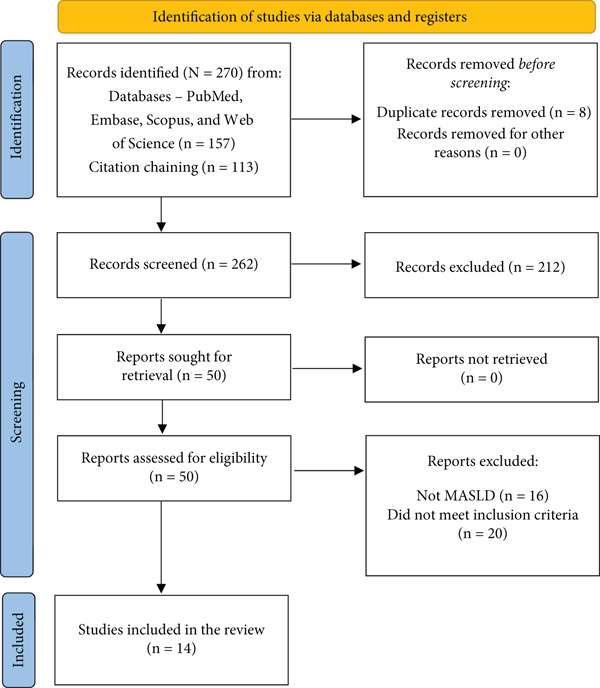
PRISMA flow diagram. Flow diagram of the study selection process, showing 157 records from databases, 113 from citation chaining (Total: 270), eight duplicates removed, 262 screened, 50 full‐text articles assessed and 14 included in the meta‐analysis [6, 8–20].

A comprehensive search was conducted across PubMed, Embase, Scopus and Web of Science using the following Boolean search terms: (“FIB‐4” OR “Fibrosis‐4”) AND (“NAFLD” OR “MASLD” OR “MAFLD”) AND (“fibrosis” OR “advanced fibrosis”) AND (“diagnostic” OR “performance” OR “accuracy”). Manual screening of the reference lists from relevant articles and reviews was also performed. The PRISMA flow diagram is detailed in Figure 1.

### 3.1. Study Selection

Two reviewers (SG and AG) independently screened titles and abstracts, followed by a full‐text review of potentially eligible studies. Discrepancies were resolved through consensus.

### 3.2. Eligibility Criteria

### 3.3. Inclusion Criteria

• Original studies evaluating the FIB‐4 score for the detection of ≥F3 in adults with MASLD.
• Use of histology or TE as the reference standard.
• Availability of sufficient data to construct 2 × 2 contingency tables (TP, FP, FN and TN).
• Use of < 1.0 or < 1.3 as FIB‐4 thresholds.
• Published in English with full‐text availability.

### 3.4. Exclusion Criteria

• Paediatric populations or patients with other chronic liver diseases (e.g., viral hepatitis and alcohol‐related liver disease).
• Non‐original articles (e.g., reviews and commentaries).
• Studies with nonstandard FIB‐4 thresholds or duplicated data (excluding less comprehensive datasets).

### 3.5. Data Extraction and Handling

Two reviewers (SG and AG) independently extracted study‐level data into a structured spreadsheet. Variables included study identifier, country/region, sample size, FIB‐4 cut‐off used, reference standard and diagnostic outcomes (TP, FP, FN and TN). Covariates such as region (India vs. global), reference modality (TE vs. histology) and threshold (< 1.0 vs. < 1.3) were recorded for subgroup and meta‐regression analyses. We extracted 2 × 2 contingency data (true positives [TPs], false positives [FPs], false negatives [FNs] and true negatives [TNs]) to ensure uniform computation of the sensitivity, specificity and FPRs across all studies. Disagreements were resolved through discussion or consultation with the authors when needed. The extracted study characteristics and diagnostic accuracy data are presented in Table 1, with subgroup sample distributions summarised in Table S1.

**Table 1:** Study characteristics and diagnostic performance metrics for included studies evaluating the FIB‐4 index for ruling out ≥F3 in MASLD.

**Study**	**N (sample size)**	**Region**	**Cut-off**	**Reference**	**TP**	**FN**	**FP**	**TN**	**Sensitivity**	**Specificity**
Chen et al. [8]	278	Global	< 1.3	TE	83	7	96	92	0.922	0.489
Caussy et al. [9]	459	Global	< 1.3	TE	49	11	170	229	0.817	0.574
Woodard et al. [10]	400	Global	< 1.3	TE	44	16	126	214	0.733	0.629
Park et al. [11]	400	Global	< 1.3	TE	71	11	83	235	0.866	0.739
Kang et al. [12]	400	Global	< 1.3	TE	90	7	92	211	0.928	0.696
Deb et al. [13]	551	India	< 1.3	Histology	107	22	85	337	0.829	0.799
Datta et al. [6]	504	India	< 1.3	Histology	64	16	101	323	0.8	0.762
Dabbah et al. [14]	337	Global	< 1.3	Histology	58	19	71	189	0.753	0.727
Damnajovska et al. [15]	200	Global	< 1.3	Histology	38	11	42	109	0.776	0.722
Hoffmann et al. [16]	350	Global	< 1.3	Histology	69	23	118	140	0.75	0.543
Anstee et al. [17]	799	Global	< 1.0	Histology	138	28	212	421	0.831	0.665
Ananchuensook et al. [18]	334	Global	< 1.0	TE	59	11	103	161	0.843	0.61
Naskar et al. [19]	388	India	< 1.0	TE	67	14	88	219	0.827	0.713
Mathew et al. [20]	121	India	< 1.3	Histology	28	7	26	60	0.8	0.698

*Note:* This table summarises individual study characteristics and 2 × 2 contingency data extracted for meta‐analysis. Region indicates geographic origin (India vs. Global). The FIB‐4 cut‐off thresholds evaluated were < 1.3 and < 1.0. Reference standards were liver biopsy (histology) or TE. Diagnostic performance metrics include TPs, FNs, FPs and TNs, from which sensitivity and specificity were calculated using standard formulas: sensitivity = TP/(TP + FN) and specificity = TN/(TN + FP). Advanced fibrosis was defined as ≥F3 on histology or the corresponding threshold on TE. These values formed the basis for pooled estimates, subgroup comparisons (region, threshold and reference standard) and meta‐regression analyses described in Section 6.

Abbreviations: FIB‐4, Fibrosis‐4 index; MASLD, metabolic dysfunction‐associated steatotic liver disease; TE, transient elastography.

### 3.6. Quality Assessment

The study quality and risk of bias were independently assessed by both reviewers using the QUADAS‐2 tool, addressing four domains: patient selection, index test, reference standard and flow/timing. All discrepancies were resolved by consensus.

### 3.7. Statistical Analysis

Pooled estimates of sensitivity, specificity and AUC were computed using a bivariate random‐effects model (Reitsma et al.) via the mada package in R.

Heterogeneity was assessed using the 95% prediction interval, whereas the proportion of variance attributable to between‐study heterogeneity was quantified using the I^2^ statistic.

Subgroup analyses were conducted for three predefined covariates:

1. FIB‐4 threshold (< 1.0 vs. < 1.3),
2. Geographic region (India vs. Global),
3. Reference standard (histology vs. TE).

These covariates were chosen a priori because they reflect clinical and methodological features that are expected to contribute to between‐study heterogeneity in FIB‐4 performance (age distribution and metabolic risk profile by region and differences in the accuracy of histology vs. TE as reference standards). These thresholds were selected a priori based on established guideline recommendations and prior meta‐analytic evidence. No post hoc threshold optimisation was performed. The biopsy‐versus‐TE comparison was conducted to address potential incorporation bias and assess the stability of performance estimates across reference standards.

Meta‐regression was performed on the logit‐transformed sensitivity and FPR using the metafor package, incorporating study‐level covariates. The standard errors were computed using the delta method.

All analyses were conducted in R (Version 4.x) using validated statistical packages, with significance defined as *p* < 0.05.

## 4. Risk of Bias and Applicability Assessment

The risk of bias was evaluated using QUADAS‐2, which assesses four domains: patient selection, index test (FIB‐4), reference standard (biopsy/TE) and flow and timing. Each domain was rated as low, high or some concerns; an overall judgement was made based on the highest risk across the domains. Two reviewers conducted the assessments, and the results are shown in Figure S1. All included studies contributed to the primary pooled analysis regardless of risk‐of‐bias rating; study quality was not used as an exclusion criterion but was evaluated to contextualise the robustness of the findings.

## 5. Statistical Analysis

The bivariate Reitsma model was used to estimate pooled sensitivity, specificity and the summary receiver operating characteristic (SROC) AUC, implemented using the mada package in R [21]. Heterogeneity was quantified using 95% prediction intervals. Subgroup analyses were stratified by region (India vs. Global), FIB‐4 cut‐off (<1.0 vs. <1.3) and reference standard (histology vs. TE). Meta‐regression was performed on logit‐transformed sensitivity and FPR using the metafor package, with variances estimated from binomial approximations [22]. Publication bias was assessed using Deeks’ funnel plot asymmetry test (log diagnostic odds ratio vs. 1/√effective sample size; *p* < 0.10 indicating asymmetry). Sensitivity analyses excluded small studies (*N* < 300), non‐< 1.3 cut‐offs and Indian cohorts. Forest plots of sensitivity and specificity were generated using the meta package, and SROC curves were visualised using ggplot2 [23, 24].

## 6. Results

### 6.1. Study Characteristics

Fourteen studies (*N* = 5521) were included, comprising 11 global and three Indian cohorts. The studies used either histology (*n* = 6) or TE (*n* = 8) as reference standards. The FIB‐4 cut‐off values were < 1.3 (*n* = 11) and < 1.0 (*n* = 3). Of the 5521 participants included, 2741 were evaluated against liver biopsy reference standards and 2780 against TE. Across FIB‐4 threshold analyses, 1021 participants were assessed using the < 1.0 cut‐off and 4500 using the < 1.3. Within TE‐based studies, 885 participants applied < 1.0 and 1895 applied < 1.3, whereas in biopsy‐confirmed cohorts, 136 applied < 1.0 and 3605 applied < 1.3. Table 1 summarises the study characteristics and diagnostic data. Detailed subgroup sample distributions by reference standard and cut‐off thresholds are provided in Table S1 to support transparency of weighting across analyses.

The proportion of cirrhosis (F4 fibrosis) could not be reported at the individual study level because the majority of included studies did not provide fibrosis‐stage–specific distributions (F3 vs. F4), instead reporting ≥F3 as a binary outcome.

### 6.2. Risk of Bias and Applicability

Risk of bias assessment using QUADAS‐2 is shown in Figure S1 (traffic light plot). Most studies had a low risk for the index test (12 out of 14 studies) but patient selection carried high or some concerns in 6 out of 14 studies due to referral‐centre cohorts. The TE‐based reference standards are associated with operator‐dependent variability. Flow and timing were low risk in 10 of 14 studies. Overall, three studies were high risk, 10 had some concerns and one had a low risk.

### 6.3. Overall Diagnostic Performance

The pooled sensitivity and specificity of FIB‐4 were 0.821 (95% CI: 0.761–0.871) and 0.619 (95% CI: 0.537–0.695), respectively. All pooled estimates reflect detection of advanced fibrosis defined as ≥F3. The SROC AUC was 0.836. Heterogeneity was evident, with prediction intervals for sensitivity ranging from 0.57 to 0.96 and for FPR from 0.21 to 0.49. The combined forest and SROC plots are shown in Figure 2. Differences in total participant numbers across stratified subgroup analyses reflect that not all studies contributed data to both threshold comparisons or both reference standards, rather than variation in overall study inclusion.

Figure 2Forest plots and summary ROC for the diagnostic performance of FIB‐4 in detecting advanced fibrosis (≥F3) in MASLD. (a) Forest plot of specificity with 95% confidence and prediction intervals; (b) Forest plot of sensitivity with 95% confidence and prediction intervals; (c) SROC curve generated using the Reitsma bivariate random‐effects model, with individual study estimates indicated by red points, and the confidence and prediction regions shown around the pooled curve. Histology‐based and TE‐based reference standards contribute separately to the pooled estimates as outlined in subgroup analyses.

**Figure:**
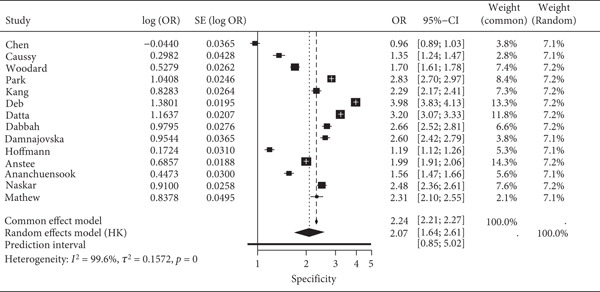
(a)

**Figure:**
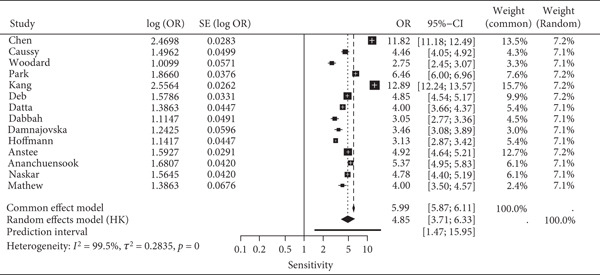
(b)

**Figure:**
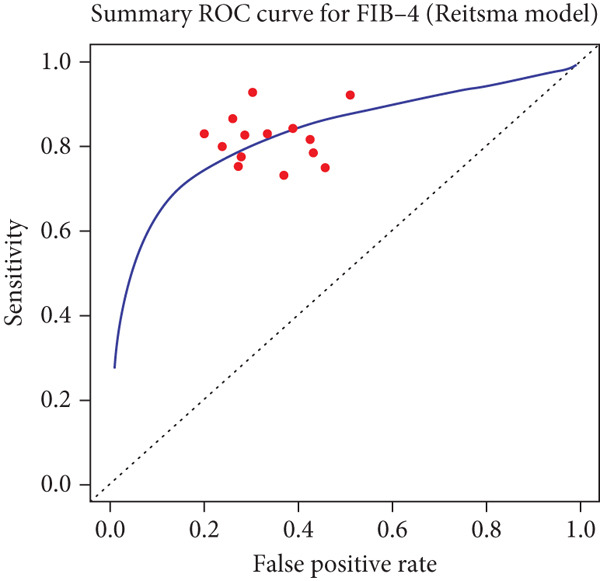
(c)

### 6.4. Subgroup Analyses

Subgroup sample sizes varied across categories due to differences in reporting between thresholds and reference standards (Table S3 provides full details).

• By region: Indian studies (*n* = 3) had a sensitivity of 0.820 (95% CI: 0.771–0.860), specificity of 0.760 (95% CI: 0.709–0.804) and AUC of 0.824. Global studies (*n* = 11): sensitivity 0.822 (95% CI: 0.778–0.860), specificity 0.636 (95% CI: 0.586–0.683) and AUC 0.798. These estimates should be interpreted cautiously due to the small number of Indian studies (*n* = 3), which may contribute to statistical instability and sampling imbalance in regional comparisons.
• By cut‐off: < 1.3 (*n* = 11): sensitivity 0.835 (95% CI: 0.792–0.870), specificity 0.642 (95% CI: 0.579–0.701) and AUC 0.822. < 1.0 (*n* = 3): sensitivity 0.743 (95% CI: 0.634–0.832), specificity 0.678 (95% CI: 0.565–0.775) and AUC 0.783.
• By reference standard: Histology (*n* = 6): sensitivity 0.798, specificity 0.707 and AUC 0.816. TE (*n* = 8): sensitivity 0.845; specificity 0.631 and AUC 0.809.

Meta‐regression did not show any significant effect of region (*p* = 0.894), reference standard (*p* = 0.064) or FIB‐4 cut‐off (*p* = 0.852) on sensitivity. For FPR, there was a nonsignificant trend toward lower FPRs in Indian cohorts (*p* = 0.062), although this finding did not reach statistical significance and should be interpreted cautiously. The reference standard and cut‐off values did not significantly influence FPR (*p* = 0.385 and *p* = 0.848, respectively). The meta‐regression results are presented in Table S2.

### 6.5. Publication Bias

Deeks′ funnel plot asymmetry test showed no significant bias (unweighted *p* = 0.5503 and weighted *p* = 0.4901). A funnel plot is shown in Figure S2.

### 6.6. Sensitivity Analyses

• Excluding small studies (*N* < 300 and *n* = 2), the pooled sensitivity was 0.818 (95% CI: 0.773–0.855), specificity was 0.665 (95% CI: 0.602–0.723) and AUC was 0.821.
• Excluding < 1.0 cut‐off studies (*n* = 3): summary estimates matched < 1.3 subgroup: sensitivity 0.818, specificity 0.665 and AUC 0.821.
• Excluding Indian studies (*n* = 3), the sensitivity was 0.822 (95% CI: 0.778–0.860), specificity was 0.636 (95% CI: 0.586–0.683) and AUC was 0.798.

The sensitivity analysis results for the pooled diagnostic sensitivity and specificity are summarised in Table S3. These results provide the basis for the subsequent interpretation of clinical relevance and implications, which are discussed below.

## 7. Discussion

### 7.1. Current Understanding

Non‐invasive fibrosis scoring tools, such as the FIB‐4 index, are recommended for initial risk assessment in MASLD, providing a practical alternative to liver biopsy. [3, 4] The FIB‐4 index′s simplicity and affordability have contributed to its widespread use, although its diagnostic accuracy varies across different populations and settings [5, 25].

### 7.2. What This Meta‐Analysis Adds

This systematic review, analysing 14 studies (*N* = 5521), synthesises FIB‐4′s performance for ruling out ≥F3 in MASLD using histology and TE. Pooled sensitivity (0.73) and specificity (0.69) at < 1.3, with an SROC AUC of 0.836, support its utility as a rule‐out test. Indian cohorts (*n* = 3) showed higher specificity (0.83) than the global average (0.66), with similar sensitivity (0.73–0.74), suggesting regional influences. Regional differences observed in subgroup analyses should be considered exploratory because only three Indian studies were available. This limited sample may exaggerate apparent differences and restrict generalisability, highlighting the need for larger, high‐quality Indian cohorts. The < 1.3 cut‐off demonstrated superior overall diagnostic performance and remains the preferred rule‐out threshold. The discussion of a lower threshold (< 1.0) in Indian settings reflects pragmatic considerations in selected populations—such as lower pretest probability of ≥F3 and health‐system constraints—rather than evidence of superior diagnostic performance. Our findings align with previous Asian cohort studies supporting the potential value of < 1.0 in populations with lower pretest probability of ≥F3, while confirming the established performance of <1.3 as the standard rule‐out threshold in broader MASLD settings. The observed regional variation in FPRs (*p* = 0.062) should be considered exploratory and hypothesis‐generating rather than confirmatory, given that it did not reach statistical significance.

### 7.3. Strengths

• Incorporating diverse reference standards (histology and TE) improves generalizability.
• Bivariate meta‐analysis with 2 × 2 data ensures robust pooling.
• Subgroup analyses clarify regional and cut‐off effects.
• Prediction intervals offer a nuanced assessment of heterogeneity.

### 7.4. Limitations

• Heterogeneity from different reference standards and settings may influence results.
• The small Indian sample (*n* = 3) limits regional conclusions. As a result, the regional comparison may primarily reflect sampling imbalance rather than true population‐level performance differences.
• Stage‐specific performance for distinguishing F3 from F4 could not be analysed because most included studies did not report separate diagnostic accuracy metrics by fibrosis stage. Future studies should evaluate stage‐stratified accuracy to better understand clinical utility in advanced versus cirrhotic disease.
• Moderate to high QUADAS‐2 bias risks (e.g., patient selection) suggest exercising caution. Although only six studies were judged to be at low risk of bias, sensitivity analyses showed that the overall diagnostic estimates remained stable, supporting the robustness of the conclusions.
• Focusing on rule‐out ability excludes consideration of positive predictive value and cost‐effectiveness.
• Performance in subgroups such as diabetics or the elderly was not examined.
• A key methodological limitation is incorporation bias due to the inclusion of TE‐based reference standards, which have lower diagnostic accuracy than liver biopsy. Although biopsy remains the gold standard, TE is increasingly used in routine clinical decision‐making, particularly where biopsy is not feasible. To mitigate this bias, we performed stratified subgroup analyses by reference standard, demonstrating comparable diagnostic performance patterns across biopsy and TE cohorts. Nonetheless, estimates within TE‐based studies may overestimate sensitivity and underestimate FPRs relative to biopsy‐verified disease, and results should therefore be interpreted cautiously.

### 7.5. Clinical implications

From a clinical perspective, these findings reinforce the role of FIB‐4 < 1.3 as a practical rule‐out tool for ≥F3 in MASLD, especially in primary and secondary care settings where biopsy or elastography may not be readily available. This should not be interpreted as a recommendation to replace the < 1.3 threshold but rather as a context‐specific adaptation in selected settings. Applying a lower threshold (< 1.0) may be considered in selected populations with lower pretest probability, such as younger metabolic cohorts or regions with lower MASLD prevalence. In practice, patients above the rule‐out threshold should be triaged to further assessment with imaging‐based elastography or liver biopsy, enabling more resource‐efficient risk stratification. Future research incorporating stage‐specific accuracy and prospective head‐to‐head comparisons across ethnic cohorts will help refine optimal cut‐off selection and enhance real‐world implementation.

## 8. Conclusion

This meta‐analysis demonstrates that the FIB‐4 index, using a threshold of < 1.3, is a reliable and pragmatic first‐line tool for ruling out ≥F3 in MASLD, with consistent diagnostic performance across diverse cohorts. Although Indian studies showed higher specificity, sensitivity remained comparable with global cohorts, and these regional differences should be interpreted cautiously given the limited number of Indian studies. Although a lower threshold (< 1.0) offers higher specificity at the cost of reduced sensitivity and may be considered in selected clinical contexts, it does not outperform the < 1.3 threshold overall. Accordingly, FIB‐4 < 1.3 should remain the default rule‐out strategy, with sequential testing using TE reserved for patients above this threshold to optimise diagnostic accuracy and resource utilisation.

## Ethics Statement

The authors have nothing to report.

## Conflicts of Interest

The authors declare no conflicts of interest.

## Author Contributions

S.G. conceptualised the study and performed statistical analysis. S.G. and A.G. conducted the literature search. A.G. drafted the manuscript, which was reviewed and verified for accuracy by S.G.

## Funding

No funding was received for this manuscript.

## Supporting information


**Supporting Information** Additional supporting information can be found online in the Supporting Information section. Figure S1: QUADAS‐2 risk of bias assessment (a) Summary plot and (b) traffic light plot. Figure S2: Deeks′ funnel plot asymmetry test for publication bias in studies evaluating the diagnostic performance of FIB‐4 for advanced fibrosis. Table S1: Meta‐regression results evaluating the impact of moderator variables on sensitivity and false positive rate (FPR). Table S2: Pooled sensitivity and specificity estimates for FIB‐4 in detecting advanced fibrosis across sensitivity analyses. Table S3: Pooled sensitivity and specificity estimates for FIB‐4 in detecting advanced fibrosis across sensitivity analyses.

## Data Availability

The data supporting the findings of this study were derived entirely from published peer‐reviewed clinical studies available in the public domain. All data used for extraction and synthesis are contained within the original articles cited in the manuscript and in Table S1. No additional raw data were generated or collected for this analysis. Therefore, data sharing is not applicable.
