# Supplementary material for: Global Bivariate Meta‐Analysis of FIB‐4 Cut‐Offs to Rule Out Advanced Fibrosis in MASLD
Source: Int J Hepatol. 2026 Jan 14;2026:9419416. doi: 10.1155/ijh/9419416 (PMC12801200; doi:10.1155/ijh/9419416)
Supplement: Supplementary file 1 — Supporting Information Additional supporting information can be found online in the Supporting Information section. Figure S1: QUADAS‐2 risk of bias assessment (a) Summary plot and (b) traffic light plot. Figure S2: Deeks′ funnel plot asymmetry test for publication bias in studies evaluating the diagnostic performance of FIB‐4 for advanced fibrosis. Table S1: Meta‐regression results evaluating the impact of moderator variables on sensitivity and false positive rate (FPR). Table S2: Pooled sensitivity and specificity estimates for FIB‐4 in detecting advanced fibrosis across sensitivity analyses. Table S3: Pooled sensitivity and specificity estimates for FIB‐4 in detecting advanced fibrosis across sensitivity analyses. [file IJH-2026-9419416-s001.docx]

**Supplementary information:**

**[A]. Supplementary Figure 1:** QUADAS-2 risk of bias assessment **(a)** Summary plot and **(b)** traffic light plot.

Each domain—**D1**: Patient selection, **D2**: Index test, **D3**: Reference standard, and **D4**: Flow and timing—was evaluated independently. Green indicates low risk of bias, yellow denotes some concerns, and red represents high risk of bias. The overall risk of bias was judged based on the highest risk observed across individual domains for each study.


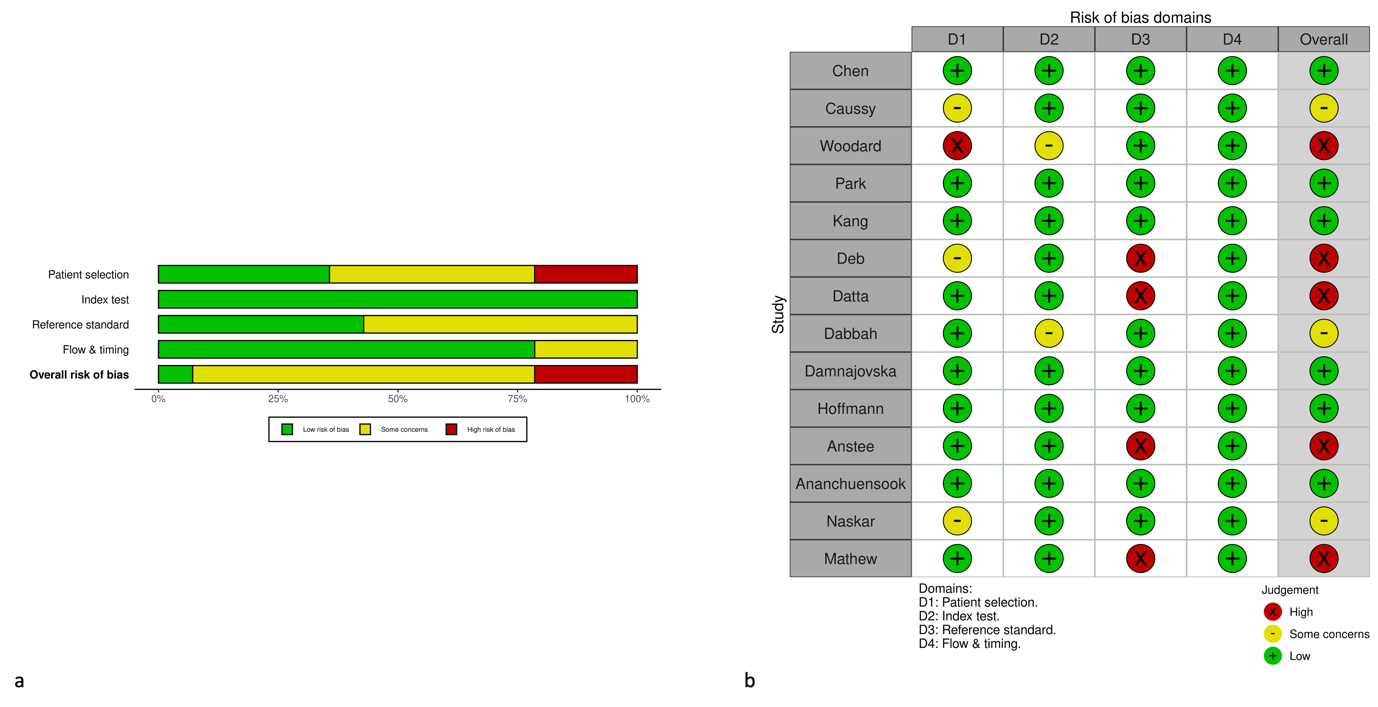


**[B]. Supplementary Figure 2:** Deeks' funnel plot asymmetry test for publication bias in studies evaluating the diagnostic performance of FIB-4 for advanced fibrosis.

The funnel plot displays the log diagnostic odds ratio (lnDOR) against the inverse square root of the effective sample size. The p-value for Deeks’ test for funnel plot asymmetry is 0.563, indicating no significant evidence of publication bias (p > 0.05).


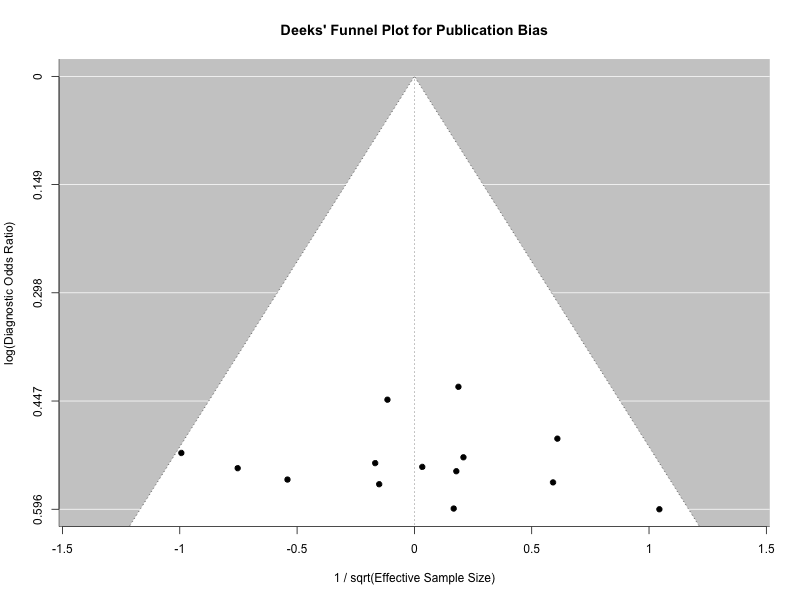


**[C]. Supplementary Table S1.** Subgroup sample sizes by reference standard, region, and FIB-4 threshold.

This table summarises participant sample sizes underlying the pooled estimates and stratified subgroup analyses. Sample totals differ across subgroup categories because individual studies contributed data to different cut-off thresholds (<1.0 vs <1.3) and to different reference standard analyses (histology vs transient elastography, TE). These numbers support transparency of weighting and help contextualise subgroup contrasts, particularly regional comparisons where only three Indian studies were available. Abbreviations: TE = transient elastography.

| **Category** | **Total Studies (n)** | **Total Participants (N)** | **<1.0 Threshold (n, N)** | **<1.3 Threshold (n, N)** |
| --- | --- | --- | --- | --- |
| All included studies | 14 | **5,521** | 3 studies, **1,021 participants** | 11 studies, **4,500 participants** |
| Reference Standard: Histology | 6 | **2,741** | 1 study, **136 participants** | 5 studies, **2,605 participants** |
| Reference Standard: TE | 8 | **2,780** | 2 studies, **885 participants** | 6 studies, **1,895 participants** |
| Region: India | 3 | **336** | 1 study, **67 participants** | 2 studies, **269 participants** |
| Region: Global | 11 | **5,185** | 2 studies, **954 participants** | 9 studies, **4,231 participants** |

**[D]. Supplementary Table S2: Meta-regression results evaluating the impact of moderator variables on sensitivity and false positive rate (FPR).**
Moderator variables assessed include region (India vs. global), reference standard modality (transient elastography [TE] vs. histology), and FIB-4 cut-off (<1.0 vs. <1.3). Results are presented as regression estimates with standard errors (SE), p-values with 95% confidence intervals (CI), and prediction intervals (PI) for both sensitivity and FPR models.

| Moderator Variable | Sensitivity (Estimate) [SE] | Sensitivity (p-value) [95% CI] | FPR (Estimate) [SE] | FPR (p-value )[95% CI] | Prediction Interval (Sensitivity) | Prediction Interval (FPR) |
| --- | --- | --- | --- | --- | --- | --- |
| Region (India vs Global) | +0.038 [0.285] | 0.894 [–0.520 to +0.595] | –0.423 [0.227] | 0.062 [–0.867 to +0.021] | 0.630 – 0.780 | 0.202 – 0.340 |
| Reference (TE vs Histology) | +0.483 [0.261] | 0.064 [–0.028 to +0.993] | +0.180 [0.208] | 0.385 [–0.227 to +0.587] | 0.701 – 0.846 | 0.188 – 0.332 |
| Cut-off (<1.0 vs <1.3) | –0.056 [0.301] | 0.852 [–0.647 to +0.534] | +0.046 [0.240] | 0.848 [–0.425 to +0.517] | 0.618 – 0.774 | 0.156 – 0.308 |

**[E]. Supplementary Table S3:** Pooled sensitivity and specificity estimates for FIB-4 in detecting advanced fibrosis across sensitivity analyses.

This table summarizes the results of three subgroup analyses: (1) excluding studies with small sample size (N < 300), (2) excluding studies using a cut-off <1.0, and (3) excluding studies conducted in India. Each analysis reports the pooled sensitivity and specificity with corresponding 95% confidence intervals derived from the madad() model.

| Study | Sensitivity | Specificity | Subset |  |
| --- | --- | --- | --- | --- |
| Caussy | 0.82 [0.72, 0.91] | 0.57 [0.53, 0.62] | Excluding N < 300 | |
| Woodard | 0.73 [0.62, 0.85] | 0.63 [0.58, 0.68] | Excluding N < 300 | |
| Park | 0.87 [0.79, 0.94] | 0.74 [0.69, 0.79] | Excluding N < 300 | |
| Kang | 0.93 [0.88, 0.98] | 0.70 [0.64, 0.75] | Excluding N < 300 | |
| Deb | 0.83 [0.76, 0.89] | 0.80 [0.76, 0.84] | Excluding N < 300 | |
| Datta | 0.80 [0.71, 0.89] | 0.76 [0.72, 0.80] | Excluding N < 300 | |
| Dabbah | 0.75 [0.66, 0.85] | 0.73 [0.67, 0.78] | Excluding N < 300 | |
| Hoffmann | 0.75 [0.66, 0.84] | 0.54 [0.48, 0.60] | Excluding N < 300 | |
| Anstee | 0.83 [0.77, 0.89] | 0.67 [0.63, 0.70] | Excluding N < 300 | |
| Ananchuensook | 0.84 [0.76, 0.93] | 0.61 [0.55, 0.67] | Excluding N < 300 | |
| Naskar | 0.83 [0.74, 0.91] | 0.71 [0.66, 0.76] | Excluding N < 300 | |
| Chen | 0.92 [0.87, 0.98] | 0.49 [0.42, 0.56] | Excluding FIB-4 <1.0 | |
| Caussy | 0.82 [0.72, 0.91] | 0.57 [0.53, 0.62] | Excluding FIB-4 <1.0 | |
| Woodard | 0.73 [0.62, 0.85] | 0.63 [0.58, 0.68] | Excluding FIB-4 <1.0 | |
| Park | 0.87 [0.79, 0.94] | 0.74 [0.69, 0.79] | Excluding FIB-4 <1.0 | |
| Kang | 0.93 [0.88, 0.98] | 0.70 [0.64, 0.75] | Excluding FIB-4 <1.0 | |
| Deb | 0.83 [0.76, 0.89] | 0.80 [0.76, 0.84] | Excluding FIB-4 <1.0 | |
| Datta | 0.80 [0.71, 0.89] | 0.76 [0.72, 0.80] | Excluding FIB-4 <1.0 | |
| Dabbah | 0.75 [0.66, 0.85] | 0.73 [0.67, 0.78] | Excluding FIB-4 <1.0 | |
| Damnajovska | 0.78 [0.66, 0.89] | 0.72 [0.65, 0.79] | Excluding FIB-4 <1.0 | |
| Hoffmann | 0.75 [0.66, 0.84] | 0.54 [0.48, 0.60] | Excluding FIB-4 <1.0 | |
| Chen | 0.92 [0.87, 0.98] | 0.49 [0.42, 0.56] | Excluding Indian Studies | |
| Caussy | 0.82 [0.72, 0.91] | 0.57 [0.53, 0.62] | Excluding Indian Studies | |
| Woodard | 0.73 [0.62, 0.85] | 0.63 [0.58, 0.68] | Excluding Indian Studies | |
| Park | 0.87 [0.79, 0.94] | 0.74 [0.69, 0.79] | Excluding Indian Studies | |
| Kang | 0.93 [0.88, 0.98] | 0.70 [0.64, 0.75] | Excluding Indian Studies | |
| Dabbah | 0.75 [0.66, 0.85] | 0.73 [0.67, 0.78] | Excluding Indian Studies | |
| Damnajovska | 0.78 [0.66, 0.89] | 0.72 [0.65, 0.79] | Excluding Indian Studies | |
| Hoffmann | 0.75 [0.66, 0.84] | 0.54 [0.48, 0.60] | Excluding Indian Studies | |
| Anstee | 0.83 [0.77, 0.89] | 0.67 [0.63, 0.70] | Excluding Indian Studies | |
| Ananchuensook | 0.84 [0.76, 0.93] | 0.61 [0.55, 0.67] | Excluding Indian Studies | |
